# Draft Genome Sequence of *Bacillus licheniformis* Strain YNP1-TSU Isolated from Whiterock Springs in Yellowstone National Park

**DOI:** 10.1128/genomeA.01496-16

**Published:** 2017-03-02

**Authors:** Joshua A. O'Hair, Hui Li, Santosh Thapa, Matthew B. Scholz, Suping Zhou

**Affiliations:** a Department of Agricultural and Environmental Sciences, Tennessee State University, Nashville, Tennessee, USA; b Vanderbilt Technologies for Advanced Genetics (VANTAGE), Vanderbilt University Medical Center, Nashville, Tennessee, USA

## Abstract

Novel cellulolytic microorganisms can potentially influence second-generation biofuel production. This paper reports the draft genome sequence of *Bacillus licheniformis* strain YNP1-TSU, isolated from hydrothermal-vegetative microbiomes inside Yellowstone National Park. The assembled sequence contigs predicted 4,230 coding genes, 66 tRNAs, and 10 rRNAs through automated annotation.

## GENOME ANNOUNCEMENT

As greenhouse gas (GHG) emissions and global average temperatures rise, research for alternative renewable energy has been actively increasing. Bioethanol, a likely substitute, can be produced by lignocellulolytic microorganisms as they ferment waste cellulosic biomass. According to the USDA, 1 billion tons of cellulose in the form of straw, corn stover, and wood wastes could be collected annually, thus replacing approximately 30% of gasoline consumption in the United States (1). Cellulosic biomass when used as a feed source also reduces GHG emissions by 86% when compared to petroleum (2). However, during consolidated bioprocessing (CBP), excess heat can become a limiting factor to mesophilic fermenting bacteria. Thermophilic lignocellulolytic microbes, on the other hand, are able to withstand increased temperatures during CBP and carry out optimal hydrolysis at or below 47°C (3). The heated springs and hydrothermal features of Yellowstone National Park are ideal locations to find these potentially unclassified cellulolytic thermophiles.

Five individual water samples were removed from Whiterock Springs, a hydrothermal feature along Solfatara Creek Trail (Yellowstone National Park, WY, USA) and vacuum-filtrated through 0.22-µm filters. Strips from the five filter paper samples were cut out and placed onto Difco nutrient agar and incubated at 37°C for 24 h. Areas with substantial growth around filter papers were harvested and restreaked on additional nutrient agar at 37°C for 24 h for individual colonies. The bacterial strain reported here tested positive for extracellular endoglucanase activity on 10% carboxymethyl cellulose plates under the Congo red assay (4). After positive cellulase testing, whole-genomic DNA was extracted using the GenElute Sigma Genomic DNA kit for Gram-positive strains (Sigma, CA, USA) following the protocol described previously (4).

Libraries were prepared with Illumina TruSeq DNA Nano sample kits using indexed adaptors (Illumina). Pooled libraries were subjected to 150-bp paired-end sequencing according to the manufacturer’s protocol (Illumina HiSeq3000). Bcl2fastq2 conversion software (Illumina) was used to generate demultiplexed FastQ files. This work was performed at the Vanderbilt Technologies for Advanced Genomics (VANTAGE) at Vanderbilt University (Nashville, TN, USA). Raw reads were then trimmed to remove bases of average below Q ≤ 3 using the Burrows–Wheeler alignment method (5). *De novo* assembly was performed using SPAdes version 3.7.1 (6) with default parameters and the “-careful” flag. The draft genome was assembled into 66 contigs with a total genome size of 4,280,972 bp (*N*_50_, 253,807) and a G+C content of 45.9%. From the 66 contigs, 39 scaffolds (nine unlocalized) were constructed through a multiple-genome alignment of YNP1-TSU and its closest neighbor *Bacillus licheniformis* ATCC 14580 (obtained through MegaBLAST against the NT/NR database) with progressiveMAUVE (7). Scaffold annotation was performed with the NCBI Prokaryotic Genome Annotation Pipeline (PGAP) version 3.3 and yielded 4,383 genes, 4,230 of which were coding genes, followed by 66 tRNAs and 10 rRNAs. With respect to the YNP1-TSU genome, several predicted cellulolytic genes (e.g., endoglucanase, cellulobiase) contained the cellulose-binding domains CBM X2 and CBM 3 (pfam03442, pfam00942) (8), suggesting that this strain implements a cellulosome mechanism when degrading cellulose (9). Further research to examine the cellulolytic activity of individual enzymes could greatly benefit second-generation bioethanol producers.

### Accession number(s).

This whole-genome shotgun project has been deposited at DDBJ/EMBL/GenBank under the accession number MIGE00000000. The version described in this paper is the first version, MIGE01000000.
